# A New Cattle Pest

**Published:** 1890-02

**Authors:** 


A New Cattle Pest.—In the July, 1889, number of the *American Naturalist*, Mr. S. W. Williston states that he has received from Prof. Cope specimens of a fly taken from cattle belonging to Mr. Thomas Sharpless, of West Chester, Pa. They were observed resting on the horns, near the base, when not feeding, having the appearance at a short distance of small patches of foreign matter. The horns were merely a resting place, the individual flies feeding upon the blood of the animals concealed in the hair along the flanks. This is the first record of the introduction from Europe of a cattle pest that bids fair to extend itself over the whole United States and be as troublesome as its nearly related pest, the stable or cattle-fly, also of European origin. The fly in question has been identified as the *Hæmatobia serrata*, or horn-fly. It will be distinguished from the common cattle-fly by its smaller size and more especially by its long palpi. Its eggs are deposited in fresh cow manure, and only twelve days are required for the insect to acquire its adult condition.

